# Somatic Mutational Profile of High-Grade Serous Ovarian Carcinoma and Triple-Negative Breast Carcinoma in Young and Elderly Patients: Similarities and Divergences

**DOI:** 10.3390/cells10123586

**Published:** 2021-12-20

**Authors:** Pedro Adolpho de Menezes Pacheco Serio, Gláucia Fernanda de Lima Pereira, Maria Lucia Hirata Katayama, Rosimeire Aparecida Roela, Simone Maistro, Maria Aparecida Azevedo Koike Folgueira

**Affiliations:** Centro de Investigação Translacional em Oncologia, Departamento de Radiologia e Oncologia, Instituto do Cancer do Estado de Sao Paulo, Hospital das Clinicas HCFMUSP, Faculdade de Medicina, Universidade de Sao Paulo, Sao Paulo 01246-000, Brazil; pedro.serio@fm.usp.br (P.A.d.M.P.S.); glaucialimap@usp.br (G.F.d.L.P.); maria.katayama@fm.usp.br (M.L.H.K.); r.roela@fm.usp.br (R.A.R.); simone.maistro@hc.fm.usp.br (S.M.)

**Keywords:** triple-negative breast cancer, high-grade serous ovarian carcinoma, somatic, young adult

## Abstract

Background: Triple-negative breast cancer (TNBC) and High-Grade Serous Ovarian Cancer (HGSOC) are aggressive malignancies that share similarities; however, different ages of onset may reflect distinct tumor behaviors. Thus, our aim was to compare somatic mutations in potential driver genes in 109 TNBC and 81 HGSOC from young (Y ≤ 40 years) and elderly (E ≥ 75 years) patients. Methods: Open access mutational data (WGS or WES) were collected for TNBC and HGSOC patients. Potential driver genes were those that were present in the Cancer Gene Census—CGC, the Candidate Cancer Gene Database—CCGD, or OncoKB and those that were considered pathogenic in variant effect prediction tools. Results: Mutational signature 3 (homologous repair defects) was the only gene that was represented in all four subgroups. The median number of mutated CGCs per sample was similar in HGSOC (Y:3 vs. E:4), but it was higher in elderly TNBC than it was in young TNBC (Y:3 vs. E:6). At least 90% of the samples from TNBC and HGSOC from Y and E patients presented at least one known affected TSG. Besides *TP53*, which was mutated in 67–83% of the samples, the affected TSG in *TP53* wild-type samples were *NF1* (yHGSOC and yTNBC), *PHF6* (eHGSOC and yTNBC), *PTEN*, *PIK3R1* and *ZHFX3* (yTNBC), *KMT2C*, *ARID1B*, *TBX3,* and *ATM* (eTNBC). A few samples only presented one affected oncogene (but no TSG): *KRAS* and *TSHR* in eHGSOC and *RAC1* and *PREX2* (a regulator of *RAC1*) in yTNBC. At least ⅔ of the tumors presented mutated oncogenes associated with tumor suppressor genes; the Ras and/or PIK3CA signaling pathways were altered in 15% HGSOC and 20–35% TNBC (Y vs. E); DNA repair genes were mutated in 19–33% of the HGSOC tumors but were more frequently mutated in E-TNBC (56%). However, in HGSOC, 9.5% and 3.3% of the young and elderly patients, respectively, did not present any tumors with an affected CGC nor did 4.65% and none of the young and elderly TNBC patients. Conclusion: Most HGSOC and TNBC from young and elderly patients present an affected TSG, mainly TP53, as well as mutational signature 3; however, a few tumors only present an affected oncogene or no affected cancer-causing genes.

## 1. Introduction

Most cancers, including breast and ovarian cancers, are mainly detected in elderly people. As a matter of fact, SEER data indicate that the peak age incidences for breast and ovarian cancers are between 65–74 and 75+, respectively. Besides that, breast cancer is the main cause of death among young women (18–40 years old). In young people who are below 40 years of age, the incidence trends for breast cancer increased in the period of 2000–2016, while it was stable or decreasing for the age groups ≥40 years during the same period. Among breast cancer subtypes, triple-negative breast cancer (TNBC) is relatively more frequent in younger patients than it is in older patients [1]. For TNBC, the overall and disease-free survival tends to be compromised in young adults compared to elderly patients [2,3].

Although the most common type of ovarian cancer in all age groups is epithelial cancer, specifically in young women, ovarian carcinoma is not common and is surpassed by borderline tumors as well as by germ cell tumors [4,5]. For those young women who are diagnosed with ovarian carcinoma, the most prevalent subtype is low-grade serous carcinoma, which is in contrast with older women, in whom the most prevalent subtype is high-grade serous ovarian carcinoma (HGSOC) [6].

In young women who are diagnosed with ovarian carcinoma at age 40 or below, the serous subtype is detected in 14.6% to 34% of the cases [7,8,9,10]; high-grade carcinoma is detected in 19% of cases; and metastatic disease is detected in 34.8% of the cases. This is in clear contrast with older patients, in whom metastatic and high-grade disease is detected in 77.5% and 67.7% of the cases, respectively [11]. These observations, which are associated with differences in disease management and secondary comorbidities in older patients, translate in a survival advantage for very young women, with the 5-year disease-specific survival estimates at 78.8% vs. 35.3% for younger and older women, respectively [11].

The main risk factor for cancer development is older age, which is due to the accumulation of DNA mutations that occur during a person’s lifetime. Cancer diagnosis at younger ages is not expected, excluding the diagnosis of cancers that are associated with hereditary syndromes [1].

Around 5% of all breast carcinoma patients and 12 to 18% of breast cancer in young patients are associated with Hereditary Breast and Ovarian Cancer Syndrome, which are caused by *BRCA1* or *BRCA2* mutations [12,13]. In TNBC specifically, a higher fraction of patients, varying from 9 to 18%, are BRCA1 (more frequently)*,* but are also BRCA2 mutation carriers [14,15]. Although not frequently, *BRCA1* somatic variants are also seen in 1–6% of TNBC tumors [16,17,18].

In ovarian carcinoma patients, more frequently, *BRCA1* mutations, but also *BRCA2* germline mutations are detected in 18 to 23% of cases [19,20,21]. In concordance, among young patients with ovarian carcinoma who are below 40 years of age, 22% are *BRCA1* mutation carriers [22]. In addition, *BRCA1* somatic variants may be detected in 8–10% of the tumor samples [19,23].

TNBC and HGSOC are both aggressive malignancies that share some similarities, such as “BRCAness”, which is defined as a defect in double-strand break repair by homologous recombination repair (HRR), which mimics the loss of function of *BRCA1* or *BRCA2* [24]. This characteristic enables patients to benefit from platinum therapy as well as from poly (ADP-ribose) polymerase (PARP) inhibitor therapy [25,26,27,28]. In fact, PARP inhibitors have shown encouraging results in the treatment of TNBC and ovarian carcinoma [29,30,31]. In addition, both TNBC and HGSOC tumor samples have been shown to be highly affected by somatic *TP53* mutations.

In summary, cancer in younger age groups is not common and may exhibit differences from the cancer that occurs in older age groups. Hence, one of our aims was to compare the somatic mutations in TNBC and HGSOC from young and elderly patients. On the other hand, TNBC and HGSOC may share some similarities in the processes that occur during tumorigenesis. Thus, another aim was to further compare the somatic characteristics of these two types of cancer, as well as to evaluate which were the potential cancer driver genes in tumors from young and elderly HGSOC and TNBC patients.

## 2. Methods

This study focused on somatic mutations. Studies focusing on molecular investigation and that were based on sequencing technology were searched in the Catalogue of Somatic Mutations in Cancer (COSMIC; https://cancer.sanger.ac.uk/cosmic; accesed on 1 May 2020), CbioPortal (https://www.cbioportal.org/; accessed on 1 May 2020), and PubMed.

The inclusion criteria were I—the diagnosis of TNBC or HGSOC; II—young (≤40 y); and III—the availability of whole-genome (whole genome sequencing—WGS) or exome (whole-exome sequencing—WES) data.

For TNBC, data from the following manuscripts were evaluated: Shah SP. et al., *Nature*, 2012; the Cancer Genome Atlas Network, *Nature*, 2012; and Nik-Zainal S. et al., *Nature*, 2016 (identified in CbioPortal); Kan Z. et al., *Nature Communications*, 2018 (identified in literature); and Banerji S. et al., *Nature*, 2012 (identified in COSMIC) (*n* = 86 patients). These studies matched the inclusion criteria [25,32,33,34,35]. Mutational and clinical data from two of the five studies (Cancer Genome Atlas Network, *Nature*, 2012; Nik-Zainal S. et. al., *Nature*, 2016) were downloaded from the International Cancer Genome Consortium—ICGC data portal (https://dcc.icgc.org/; release date: 1 January 2020). The other studies had their data downloaded from the supplementary data from their respective original articles.

For HGSOC, only one study was evaluated (Cancer Genome Atlas Network, *Nature*, 2011) since this was the only one to fulfill the inclusion criteria [36] at the time of analysis.

The studies that were chosen according to the inclusion criteria were also used to select data from patients who were aged ≥75 year for further comparison between age groups.

Since the variant data were obtained from both WES and WGS sequencing, we first analyzed the normalized data by excluding non-coding variants. Silent variants were also filtered out, as this information was absent in some studies. Variant data were annotated with Oncotator (http://portals.broadinstitute.org/oncotator/; v1.5.3.0; accessed on 1 May 2020) to determine the genomic position, pathogenicity prediction through in silico tools, amino acid change, and other additional data.

For the interpretation of the gene variants, previous literature was consulted for relevant criteria (Sukhai MA. et. al., 2016 and Li MM. et. al., 2017) [37,38], but since the somatic data were mainly classified according to clinical actionability, which could limit the present analysis, we adopted a less restrictive classification model at first in order to explore possible driver genes in the cancer types that were evaluated. The classification criteria are detailed below.

For a gene presenting a variant, we collected data from/about:

1—Cancer Gene Census database (CGC; v.89), which is a curated repository of mutated genes that are causally implicated in cancer. Both tier 1 and tier 2 genes were considered to be relevant.

2—Candidate Cancer Gene Database (CCGD) [39] which contains data about multiple studies that have investigated the contribution of certain genes to mice tumorigenesis based on mutational insertion transposons studies. For the latter database, we only considered those genes within the higher rank classification (rank A).

3—Potentially pathogenic variants: truncated somatic variants (frameshift, nonsense, and canonical splice-site ±3).

4—Possibly pathogenic variants: missense somatic variants were then assessed using variant functional impact prediction tools: SIFT (https://sift.bii.a-star.edu.sg/, accessed on 1 May 2020); Polyphen-2 (http://genetics.bwh.harvard.edu/pph2/, accessed on 1 May 2020); FATHMM (http://fathmm.biocompute.org.uk/, accessed on 1 May 2020); Mutation Taster (http://www.mutationtaster.org/); and Mutation Assessor (http://mutationassessor.org/r3/), accessed on 1 May 2020. Missense variants were considered to be possibly pathogenic if they accomplished one of the following items: I—the variant was classified as pathogenic in 3 out of 5 predictors; II—the missense variant was placed in a CGC gene and was classified as pathogenic in at least 1 of 5 of the prediction tools

5—Clinical and biological evidence: Finally, we explored all the variants from the CGC oncogenes and TSGs using the OncoKB Database (https://www.oncokb.org/; accessed on 2 November 2021). The database contains curated clinical and biological evidence at the variant and gene level and reports oncogenic and mutation effects from functional and clinical studies as well as biomarkers with data approved by the FDA and other professional guidelines with compelling evidence. Variants were classified as: I—likely loss-of-function if there was functional evidence in the literature; II—likely gain-of-function if there was functional evidence in the literature; III—loss-of-function if there was functional evidence in literature and/or clinical evidence and/or other therapeutic/diagnostic/prognostic/FDA levels (as described in https://www.oncokb.org/levels; accessed on 2 November 2021); IV—gain-of-function if there was functional evidence in the literature and/or clinical evidence and/or others therapeutic/diagnostic/prognostic/FDA levels (as described in https://www.oncokb.org/levels; accessed on 2 November 2021); V—likely neutral if there was biological and/or clinical evidence in the literature; VI—inconclusive if functional and/or clinical assays were inconclusive; VII—not reported if the gene or variant was not found or curated in the literature [40].

6—DNA repair related genes: A list of genes involved in DNA repair pathways was assembled according to the following published manuscripts: Lange SS, Takata K, Wood RD, *Nature Reviews Cancer*, 2011; Wood RD, Mitchell M, Sgouros JG, Lindahl T, *Science*, 2001; and Chae et al., *Oncotarget*, 2016. This list of genes was used to look for affected genes that are involved in the different DNA repair pathways [41,42,43].

The Maftools 2.2.0 package [44] and built-in R packages were used to generate frequency summaries for variant types and mutational signatures. The trinucleotideMatrix, estimateSignatures and extractSignatures functions from the Maftools package were used for the mutational signatures analysis based on the Alexandrov publication [45]. The methodology is available at https://github.com/PoisonAlien/maftools (accessed on 1 June 2020).

Gene ontology analyses were performed through g:Profiler (https://biit.cs.ut.ee/gprofiler_archive2/r1760_e93_eg40/web/; accessed on 1 June 2020). Redundant gene ontology terms were filtered out with Revigo (http://revigo.irb.hr/; accessed on 1 June 2020) [46,47].

Descriptive and inferential analyses for tumor and age comparison were conducted in R.v.3.5.0 (Rstudio v.1.1.453). The Mann–Whitney U test was used to compare continuous variables between the groups and Fisher’s Exact test for the categorical ones.

Germline data were not evaluated because they were not available as open access data.

## 3. Results

Mutational data were retrieved from the literature and public databases for 21 young and 60 elderly HGSOC patients as well as for 86 young and 23 elderly TNBC patients.

Among the HGSOC patients, the median age at diagnosis was 37 and 78 years for the young and elderly groups, respectively. Most patients were diagnosed at clinical stages III and IV (95% young vs. 88% elderly). For TNBC patients, the median age at diagnosis was 36 and 80 years for the young and elderly age groups, respectively, and around half of the patients in both age groups presented with disease clinical stage III.

At first, we focused on the number and profile of genes that were affected by non-synonymous coding variants, independent of whether they were neutral or potentially pathogenic/possibly pathogenic. Subsequently, we analyzed the number and profile of driver mutations in genes that were catalogued in the Cancer Gene Census (CGC) database. We also looked forward to identifying possibly putative drivers, which were represented by affected genes that were catalogued in the Candidate Cancer Gene Database (CCGD) with supportive cancer-related literature.

### 3.1. Base Substitutions

**Figure 1:**
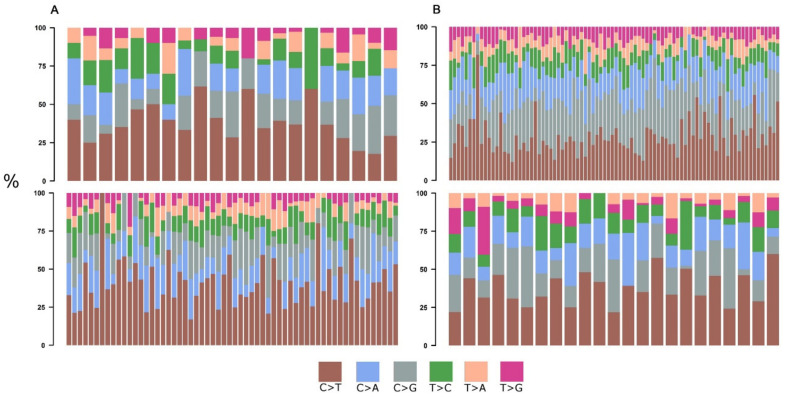
Single base substitutions. (**A**) Distribution of single base substitutions across young (**top**) and elderly (**bottom**) HGSOC patients. (**B**) Distribution of single base substitutions across young (**top**) and elderly (**bottom**) TNBC patients.

For HGSOC as well as for TNBC, the most prevalent base substitutions in both age groups were C>T (HGSOC: 37% young vs. 41% elderly; TNBC: 27% young vs. 35% elderly) (Figure 1A,B; Table 1 and Table S1). There was no difference in the frequency of transitions (TI) between young and elderly HGSOC patients, as well as in transversions (TV). In TNBC, the frequency of TV in young patients was higher than in the elderly ones. Also, young patients had a higher frequency of TV in comparison to TI (TI: 38%; TV: 62%) (Table S2).

### 3.2. Mutational Signatures

**Figure 2:**
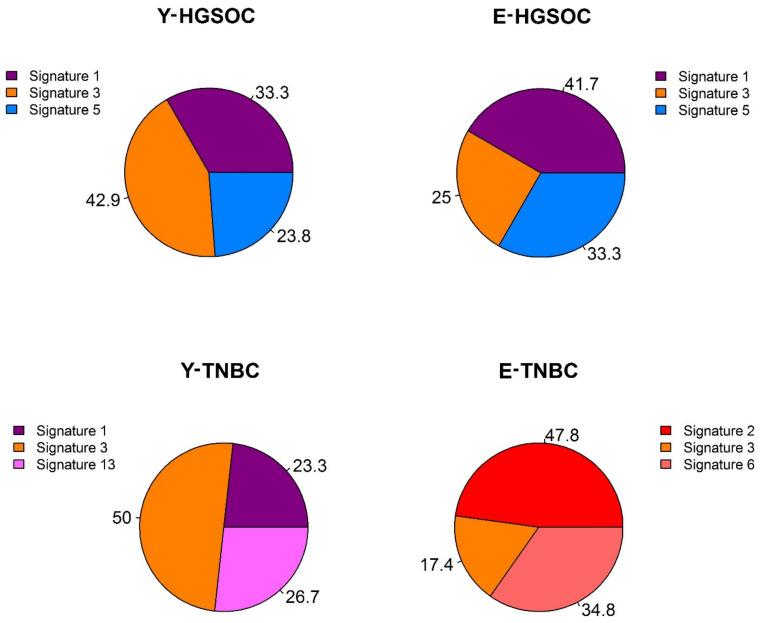
Mutational signatures: The pizza plots show the distribution (%) of the signatures across age groups in both cancer types. Y-HGSOC: young HGSOC patients; E-HGSOC: elderly HGSOC patients; Y-TNBC: young TNBC patients; E-TNBC: elderly TNBC patients.

In HGSOC, the most frequently detected mutational signatures in the young and elderly cohorts of patients were signatures 1 (spontaneous deamination of 5-methylcytosine due to age), 3 (defects in double-strand break repair by homologous recombination), and 5 (unknown etiology). Hence, HGSOC tended to present a more homogeneous pattern of mutational signatures in both age groups (Figure 2 and Figure S1).

On the other hand, in TNBC, the most frequent mutational signatures in young and elderly patients seemed more heterogeneous, as only signature 3 appeared in both age groups. The other most frequent mutational signatures that were identified in young people were signatures 1 and 13, and in elderly people, the most frequently occurring signatures were signatures 2 and 6 (Figure 2; Table 1; Figure S1). It is interesting to observe that the APOBEC cytidine deaminase signatures were significantly detected in both young (signature 13) and elderly people (signature 2), but following different strand *bias*, the APOBEC signature in young patients was correlated with transversions, and the one in elderly patients had transitions, which may explain the previously mentioned higher frequency of transversions in TNBC from the young age group. On the other hand, signature 1, which is related to age, was present in tumors from young people, and signature 6, which is related to defective DNA mismatch repair, was present in tumors from elderly people.

Hence, in both the HGSOC and TNBC samples, the only mutational signature that was significantly detected in young as well as in elderly patients was signature 3, indicating that BRCAness is an important feature in cancers occurring at both extremes of age and in both tumor types. In contrast, each tumor type presented an exclusive mutational signature pattern, regardless of age: mutational signature 5 was more characteristic of HGSOC and APOBEC-related signatures of TNBC.

### 3.3. Number of (Non-Synonymous) Coding Variants

In the HGSOC samples, the median number of non-synonymous coding variants per sample was 37 for young and 44 for elderly women; for TNBC, it was 50.5 for young and 61 for elderly women. There were no significant differences when the samples from the young and elderly patients from both tumor types were compared. However, when comparing the TNBC and HGSOC samples from the same age groups, TNBC presented a higher number of coding variants compared to HGSOC (Table 1).

Compared to the whole cohort of TCGA samples (including all ages and tumor subtypes), both the young and elderly HGSOC groups showed a lower number of coding variants per sample (TCGA OV cohort vs. young HGSOC, *p* = 4.3 × 10^−6^; TCGA OV cohort vs. elderly HGSOC, *p* = 1.8 × 10^−5^) (Figure S2; Table S3). In contrast to HGSOC, both young and elderly TNBC patients showed a higher number of coding mutations when compared to the complete TCGA Breast Cancer (BRCA) cohort (TCGA BRCA cohort vs. young TNBC, *p* = 0.019; TCGA BRCA cohort vs. elderly TNBC, *p* = 0.0091) (Figure S2; Table S3).

**Figure 3:**
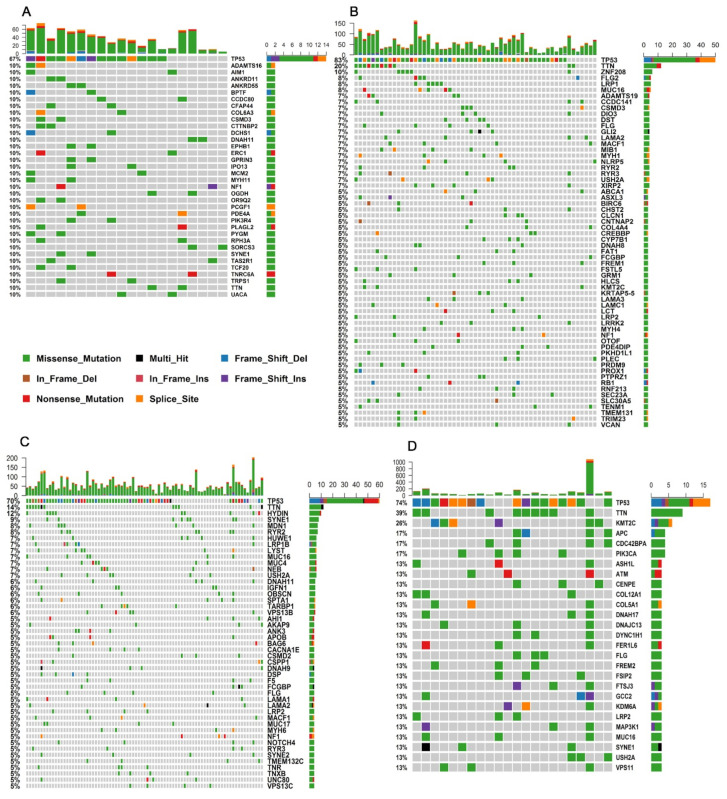
Most frequently affected genes: (**A**) Young HGSOC patients (20 samples shown); (**B**) elderly HGSOC patients; (**C**) young TNBC patients; (**D**) elderly TNBC patients. Each column represents a patient, and each line represents a gene.

We also evaluated the number of coding variants per sample according to *TP53* status. In HGSOC, 7 out of 21 (33%) tumors from young patients and 10 out of 60 (16%) tumors from elderly patients were positive for *TP53*wt, but no difference in coding variants per sample according to TP53 status was observed (*p* = 0.12) (Figure 3A,B; Table S4). For TNBC, the patients with affected *TP53* showed a higher number of variants per sample (*p* = 0.012) (Figure 3C,D; Table S4).

Considering the median number of CGC per sample, there was no difference between the young and elderly HGSOC patients (*n* = 3, young vs. *n* = 4, elderly; ns). In contrast, a higher number of CGC per sample was detected in the elderly patients in the TNBC group (*n* = 3, young vs. *n* = 6, elderly; *p* = 0.00013) (Table 1).

### 3.4. Genes Most Commonly Affected

For this analysis, all genes presenting a coding variant, independent of the gene function or the effect of the variant, were considered (Table S4).

In total, 190 samples from young and elderly HGSOC and TNBC were analyzed. The 50 most commonly affected genes were present in 93% (177/190) of all of the samples (affected in at least six samples of all four groups added together), including 18 genes (36%) that were shown to be affected at least once in each one of the four groups: *TP53*, *TTN*, *SYNE1*, *USH2A*, *CSMD3*, *MACF1*, *MUC17*, *TARBP1*, *TENM1*, *VPS13B*, *DYNC1H1*, *F5*, *FSIP2*, *HMCN1*, *MXRA5*, *PLEC*, *DNAH8*, *and UNC79* (Figure S3).

As expected, the most frequently altered gene was *TP53*, which was affected in 67% and 83% of the young and elderly HGSOC patients and in 70% and 74% of the young and elderly TNBC samples, respectively.

The second most frequently affected gene was *TTN* (18.4% of samples), followed by *MUC16* and *USH2A* (7.4%; 14 out of 190 samples). *TTN* and *MUC16* code for two of the longest known proteins in the human genome, with 35,992 and 14,508 amino acids, respectively, and are frequently affected in a high percentage of tumor types, i.e., 24.49% (*TTN*) and 15.21% (MUC16) (COSMIC database, available at https://cancer.sanger.ac.uk/cosmic/gene/analysis; accessed on 27 May 2020).

*TTN*, *MUC16,* and *USH2A,* as well as another 23 genes (out of the list of the top 50 genes), integrate a list of FrequentLy mutAted GeneS, termed FLAGS. These genes present longer protein-coding sequences and a greater number of paralogs. They also display less evolutionarily selective pressure than expected [48]. Although FLAGS have been more frequently associated with disease causality than expected for protein-coding genes in general, their functional impact should be interpreted with great care because somatic mutations without functional consequences may occur during cell division and may represent passenger mutations that do not contribute to cancer development [49]. This observation may be applied to 11 out of 26 FLAGS as well as to another three genes that were not originally classified as FLAGs (*ZNF208*, *FSIP2*, *MXRA5*), as they mainly presented missense variants with non-pathogenic effects in all effect prediction tests.

Although its long length, which is consistent with FLAGS, *MUC16* is also listed in the CGC database (Tier 2). *MUC16* codes for a mucin, which is commonly shed in ovarian cancer and less frequently in other tumors of epithelial origin. *MUC16* may transform the NIH3T3 mouse fibroblast cell line and may thus, be considered an oncogene [50].

In fact, in the list of the top 50 most frequently mutated genes from the present series, only ten genes were classified in the Cancer Gene Census database: *TP53*, *MUC16*, *CSMD3*, *KMT2C*, *NF1*, *LRP1B*, *PIK3CA*, *ATM*, *BIRC6,* and *MUC4*.

The top 20 most frequently affected genes as listed in the Cancer Gene Census (CGC) considering the 190 samples from young and elderly HGSOC and TNBC appear on Table 2.

In HGSOC, besides *TP53*, other CGCs that were affected in samples from both young and elderly patients were TSGs, such as *CSMD3* (CUB and Sushi multiple domains 3), *NF1* (neurofibromatosis type 1 gene), *CLTCL1* (clathrin; heavy polypeptide-like 1), and *RB1* (retinoblastoma); however, there were no differences in their frequencies between age groups (Table 2).

In the young cohort of HGSOC, besides *TP53*, another 35 genes were recurrently altered in at least two out of 20 samples (10%) (Figure 3A). One of these genes was *PCGF1*, or polycomb group ring finger 1. It is interesting to point out that the same potentially pathogenic variant (canonical splice site) was found in both young patients. *PCGF1* integrates the *BCL6* corepressor (BCOR) complex, which is involved in transcriptional gene silencing. Inactivating somatic mutations in BCOR were detected in patients with acute myeloid leukemia (AML) and other cancers, suggesting that it might function as a TSG [51].

In elderly HGSOC samples, other CGCs that were frequently altered were *MUC16* (detected in 8.3% of the tumors) and *BIRC6* (5%), which are classified as oncogenes, as well as *KMT2C* and *ATM* (5% each), which are classified as TSGs (Figure 3B).

Turning to the top most frequently affected CGCs in TNBC, besides *TP53*, another 15 genes were altered in samples from both age groups, including *MUC16*, *PIK3CA*, *BIRC6*, and *MUC4*, which can be classified as oncogenes, as well as *CSMD3*, *KMT2C*, and *ATM*, which can be classified as TSG, among others (Table 2, Figure 3C,D). Interestingly, *KMT2C* was more frequently altered in elderly patients, i.e., 26% (6/22) than it was in young patients, i.e., 1% (1/86) (*p* = 0.006). *KMT2C* (lysine (K)-specific methyltransferase 2C) promotes the methylation of histone H3 and regulates gene transcription, and even though it is described as TSG in medulloblastoma in COSMIC (https://cancer.sanger.ac.uk/cosmic/census?tier=all#cl_search; accessed on 25 May 2020) in TNBC specifically, it is characterized as both a tumor suppressor and an oncogene. Accordingly, in other studies, *KMT2C* alterations were correlated with more advanced ages (>50 yo) in breast cancer patients [52] and were described as being frequently altered in TNBC [52,53,54,55,56]. Nonetheless, among the top 20 CGCs, it is interesting to observe that with the exception of 1 gene (*MUC4*), all of the other 19 genes that were most frequently altered in the TNBC tumors from young patients were also affected in the HGSOC tumors from elderly patients (Table 2). This observation probably reflects the larger number of samples that was evaluated for these age groups, which allowed the characterization of a larger number of recurrently altered genes (Y TNBC *n* = 86; E HGSOC: *n* = 60).

In TNBC, after *TP53*, the most frequently altered gene in both age groups was *TTN*, which was altered in 14% and 39% of young and elderly patients, respectively.

**Figure 4:**
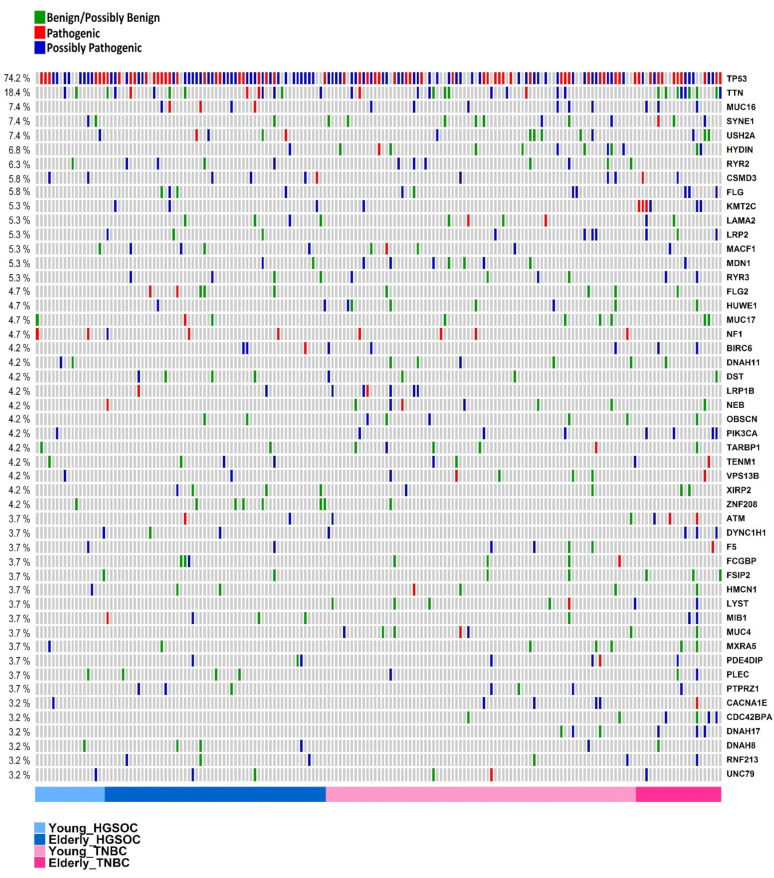
Frequently affected genes considering all samples of HGSOC and TNBC (young -Y and elderly -Y) classified according to their pathogenicity. Combined top 50 most frequently affected genes classified as potentially pathogenic (red), possibly pathogenic (blue) as described in methods, and benign (green). Each column represents a patient, and each line represents a gene (177 samples shown).

Although many different genes were altered in the whole cohort, as determined in the previous section describing the most commonly affected genes, not all of them were affected by potentially pathogenic/possibly pathogenic variants. Figure 4 (also, Figure S3 and Table S7) shows the top 50 genes that are the most frequently mutated in tumors from the four groups according to their classification as being potentially pathogenic, possibly pathogenic, or benign; for the latter category, genes other than CGC were not considered to be pathogenic in variant effect prediction tests. Examples of these genes are *MUC17*, *ZNF208*, *FSIP2,* and *MXRA5*.

On the other hand, 8 of the top 50 (16%) genes were CGCs, where all of the mutations that were detected were potentially/possibly pathogenic, including *TP53*, *MUC16*, *CSMD3*, *KMT2C*, *NF1*, *BIRC6*, *LRP1B,* and *PIK3CA*.

### 3.5. Cancer Driver Genes

Our main goal was to evaluate what the potential cancer driver genes in the tumors from young and elderly HGSOC and TNBC patients were. We assumed that cancer driver genes or cancer-causing genes were those genes that were catalogued in the Cancer Gene Census database (CGC) (accessed May 2020) that were affected by potentially pathogenic (nonsense, frameshift, and canonical splice-site) or possibly pathogenic (missense variants predicted as pathogenic in at least one out of five variant effect prediction tools) variants (Tables S4–S6).

Most *TP53* variants were present in the DNA binding domain (DBD) in the four groups of samples (Y HGSOC: 78% vs. E HGSOC: 86%; Y TNBC: 88% vs. E TNBC: 75%). Some mutations were in commonly affected hotspots of the DBD, such as the *TP53* variant *p*.R175H, which was detected in eight samples (E HGSOC: *n* = 1 (2%); Y TNBC: *n* = 6 (10%); E TNBC *n* = 1 (6%)) and *p*.R248W, which was detected in six samples (E HGSOC *n* = 4 (8%); Y TNBC: *n* = 1 (1%); E TNBC: *n* = 1 (6%). A third *TP53* variant, that was exclusively observed in young TNBC patients (*n* = 4 or 6%), was p.R213*, a nonsense variant. All of the abovementioned variants were previously reported as germline variants and were shown to be related to Li–Fraumeni syndrome [57,58,59] (Figure S4).

We then evaluated which were the cancer driver genes for each specific tumor sample were, based on the above assumptions. All of the affected oncogenes and tumor suppressor genes classified as CGCs tier 1 and tier 2 appear on Table 3. For those patients with no affected oncogene or TSG, we assumed that the genes that were catalogued in the Candidate Cancer Gene Database (CCGD) and with supportive cancer-causing related literature as putative drivers.

For the HGSOC samples, almost all variants in the CGC genes were considered to be potentially pathogenic/possibly pathogenic (based on the described criteria), except for variants in eight genes, *HIP1*, *IKBK*, *FAT1*, *PALB2*, *KIT*, *PRMD2*, *RET,* and *WNK2*, each one detected separately in one single sample. Looking for TNBC samples, the missense variants in 32 CGC genes were considered to be non-pathogenic, in accordance with the criteria exposed above, with focus on *MUC4*, which was considered non-pathogenic in four different tumors. Another gene, *CDH1*, which is considered a cancer driver for hereditary lobular breast cancer, presented a non-pathogenic variant in this cohort of TNBC samples. To further assign the mutation effect of the variants in CGC oncogenes and TSGs for the selected series of samples, we explored each variant (oncogenes and TSGs) in the OncoKB database. Detailed data are reported in Table 3.

In all four groups (HGSOC and TNBC young and elderly), most of the samples presented at least one affected oncogene in association with at least one affected tumor suppressor gene, which were represented by combinations of variants in OG and TSG, OG and a dual role gene (OG or TSG), TSG and a dual role gene, or even in at least 2 dual role genes (one might assume the role of OG and the other of TSG). This condition was verified in 66.6% and 85.0% of the young and elderly HGSOC cases as well as in 72.1% and 100% of the samples from young and elderly TNBC, respectively (Table 1). Among the associations were genes such as *KRAS* and *NF1* (Y-HGSOC), *ERBB2* and *DNMT3A* (Y-HGSOC), *PIK3CA* and *TP53* (Y-TNBC and E-TNBC), among others (Table 3).

In the young HGSOC cohort, three samples (14.3%) presented variants in the genes that were involved in the Ras and or PIK3CA signaling pathways, including *KRAS* and *PIK3CA* (each one in one single sample) and *NF1* (altered in two samples, one of them, in association with *KRAS*). In two of these samples, there was an association with *TP53* mutations. In addition, in three samples, the DNA repair genes *RAD17*, *BLM*, and *ERCC3*, were mutated, which occurred in concomitance with the *TP53* mutations. In five tumors (23.8%), only one potentially pathogenic/possibly pathogenic CGC, such as *CDKN1B* or *KAT6B*, which are considered to be TSGs (each one in a single tumor), was mutated, as was *TP53* (in three different samples), which is considered to have a dual role gene, in accordance with CGC database (https://cancer.sanger.ac.uk/cosmic/census?tier=all#cl_search; accessed on 22 May 2020) [60] (Table 3).

In elderly HGSOC, mutations in the genes that are involved in the Ras and or PIK3CA signaling pathways were detected in nine samples (15%), including the genes *KRAS* and *BRAF* (each one in 1 tumor); *PIK3CB* and *MTOR* (each one in 2 tumors); and *NF1* (in three different samples). Multiple gene mutation associations were detected. In seven tumors (11.6%) there was an association of Ras and/or PIK3CA defective signaling with mutated *TP53*. Another mutation combination was observed between *RB1* and *TP53,* which was detected in three different samples (5%). In addition, in another 12 samples from elderly HGSOC (20%), a DNA repair gene was mutated; such affected genes included *ATR*, *CDK12*, *MLH1*, *POLG*, *FEN1*, *BARD1,* and *XPC*, each one found in a single sample; *ATM*, *BRCA2,* and *FANCA*, each one found in two different samples. In one sample, there was a concomitant mutation in *FANCA* and *ATR*, and in other ten samples (16.6% of the total samples), *TP53* was concomitantly mutated with one of these genes. *MUC16* was mutated in five samples (8.3%) always in concomitance with multiple other CGCs, including TSGs, as well as *TP53*. In four tumors, a growth factor receptor such as *EGFR*, *ERBB2*, *FGFR4*, and *PDGFRA*, was mutated, in combination with other mutations in TSGs, generally in concomitance with *TP53*. Finally, in four samples, the only mutated gene was *TP53,* and in another two samples, only one oncogene was mutated (*KRAS* or *TSHR*). Nonetheless, in two samples (3.4%) mutations, not one defective CGC was detected (Table 3).

As described above, most HGSOC and TNBC patients carry a defective TP53, regardless of age onset (Y: 66.7%; E: 81.7%). Among those who do not, 23.8% of young and 6.67% of elderly patients carry alterations in other TSGs. Most of these genes are involved in epigenetic regulation (*KMT2C*, *KA6T6B*, *DNMT3,* and *DROSHA*), transcriptional regulation (*MED12*, *LZTR1*, *PHF6*, *SMARCA4*, and *TBX3*), and DNA repair (*POLG*, *ATM*, *RAD21*). Among these TSGs, *FLCN*, *PHF6,* and *TBX3* (found in elderly patients) were the only genes whose variants were reported by OncoKB as presenting likely loss-of-function effects, which might indicate new putative drivers. 

Considering TNBC from young patients, most samples (90.7%) presented at least one affected TSG according to the CGC database. Besides *TP53* (mutated in 69.8% of the samples), other gene variants that had already been classified with likely loss-of-function in the OncoKB database were *NF1* (affected in three samples or 3.5%) and *PTEN*, *PHF6*, *ZFHX3*, *PIK3R1*, *GRIN2A*, *NF2,* and *MED12* (each one affected in a single sample). The other genes cited as TSG in Table 3 are considered as TSG in the CGC database and are affected by potential damaging alterations, including *ATRX*, *BARD1+POLG; NOTCH1; PTPRC; POLE+SETD2; TRT2; ATR + SMARCA4; BAP1; CDH1 + MGMT; and AXIN1 + CBLB* (each gene, alone or in the combination shown, affected in one sample). Despite that, in eight samples (9.3%), there were no affected TSG. Moreover, in 12 samples (13.9%), only 1 CGC gene was mutated, including 2 oncogenes, PREX*1* and *RAC1*; 5 TSGs, *BAP1*, *NF1*, *PIK3R1*, *PTPRC,* and *ZFHX3*, and 2 dual role genes, *NOTCH1* and *TP53*, the latter, in three samples. Besides that, in six tumors (6.9%), a well-established cancer driver gene was not detected (Table 3).

Among TNBC from young patients, 18 out of 86 (20.9%) tumors presented mutations in genes involved in the Ras and/or PIK3CA signaling pathways, such as *AKT3*, *BRAF*, *MAP2K2*, *MAPK1*, *NF1*, *NF2*, *PTEN*, *PIK3CA*, *PIK3CB*, *PIK3R1*, *RAC1*, *RAF1,* and *RANBP2. NF1*, *PIK3CA,* and *BRAF* were mutated in four, three, and two samples, respectively. In addition, there was an association between mutated OGs and TSG, such as *AKT3* and *NF2*, and PIK3CA and NF1 in two samples. Besides that, NF1 was the sole mutated CGC in two samples, and *PIK3R1* and *RAC1* were the only mutated CGC, each one detected in a single sample. In nine of these samples, gene mutations involved in the Ras pathway were in concomitance with *TP53* mutations.

Mutations in the DNA repair genes, such as *BRCA2*, *ATM*, *BAP1*, *BARD1*, *FANCG*, *MGMT*, *MUTYH*, *POLE*, *POLG*, *POLQ*, *and RECQL4* were detected in nine tumors. *BAP1* and *BRCA2* were mutated in two tumors, in association with *FANCG* in one of the tumors. *BARD1* and *POLG* were concomitantly mutated in one tumor. In addition, in five tumors, a defective DNA repair gene was concomitant with *TP53* mutation.

Among the elderly TNBC patients, all 23 samples presented more than one affected CGC, represented by at least one mutated oncogene and one TSG. In addition, all the tumors presented gene variants classified as likely loss of function in the OncoKB database, except for one tumor. Besides TP53 (affected in 73.9% of the samples), other affected TSG were *KMT2C*, *TBX3,* and *ATM*. A dysfunction in the Ras and/or PI3K pathways was observed in 8 out of 23 samples (34.8%), among which five (21.7%) were associated with *TP53* mutation. The Ras family members were mutated in four tumors (*KRAS*, *n* = 2; *HRAS* and *NRAS*, *n* = 1); *PIK3CA* in four tumors (in concomitance with *KRAS* in two tumors), and *PTEN* in another two tumors. In addition, *BRCA1* was mutated in two tumors, and *BRCA2* was found in one tumor, all three in concomitance with *TP53* (13% of the tumors). Interestingly, all of the *KRAS* variants from both tumor types were located at the same position, a known hotspot (p.G12V) (Table 3).

Ontology analysis was performed to verify which biological processes or molecular functions were enriched for the genes that were found to harbor potentially pathogenic variants (nonsense, frameshift or canonical splice site) (HGSOC—young: 115 genes vs. elderly: 435 genes; TNBC—young: 671 genes vs. elderly: 363 genes) or through variant effect prediction tools (HGSOC—young: 156 genes vs. elderly: 538 genes; TNBC—young: 935 genes vs. elderly: 568 genes) in both age groups of HGSOC and TNBC. Processes that were enriched in the two age groups from both cancer types were cell adhesion and motility, the extracellular matrix, and ion channels (Tables S8–S11).

## 4. DNA Repair

**Figure 5:**
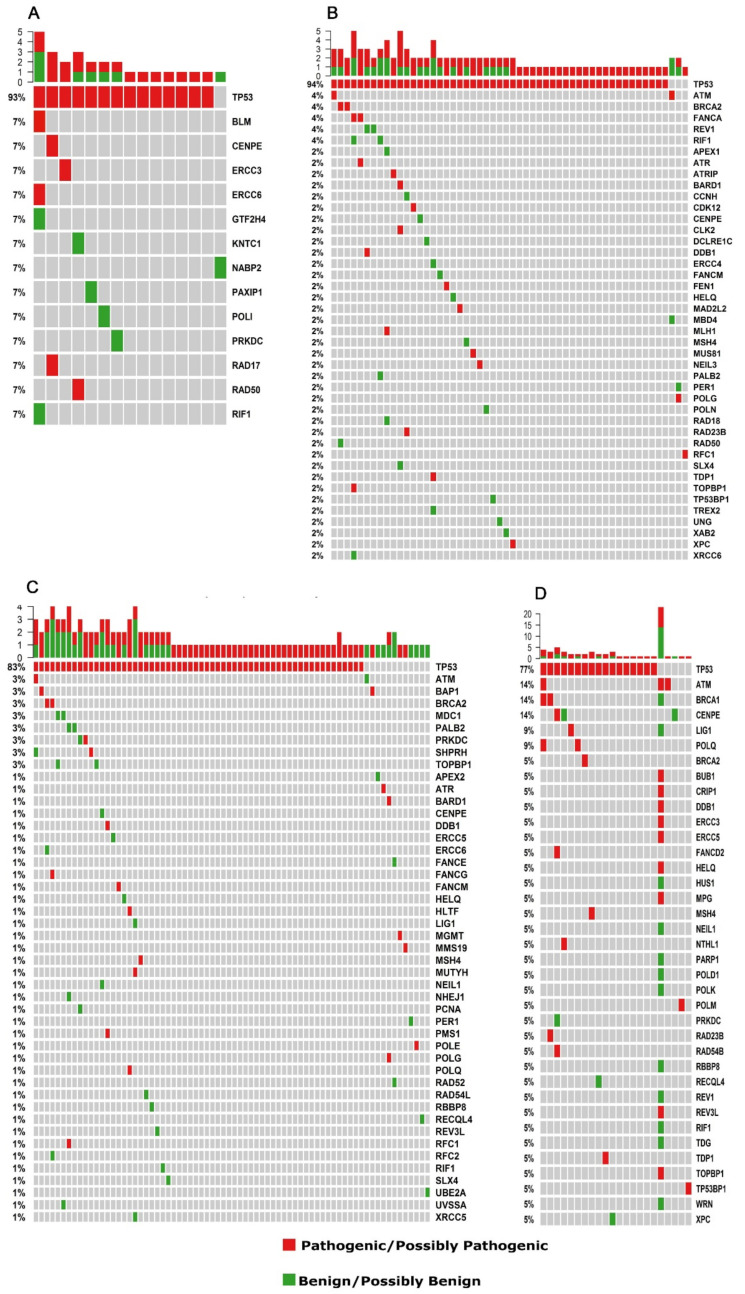
Affected DNA-repair related genes. (**A**) Young HGSOC patients; (**B**) elderly HGSOC patients; (**C**) young TNBC patients; (**D**) elderly TNBC patients. Each column represents a patient, and each line represents a gene. Potentially and possibly pathogenic variants are shown in red, and potentially and possibly benign variants are shown in green.

We then carefully searched for genes that were directly or indirectly related to DNA repair. This analysis included DNA repair genes that were not currently considered a CGC gene, thus expanding the view of the previously conducted analyses of cancer driver genes (Figure 4). Excluding *TP53* mutations, which were the most frequent mutations found in both age groups for both HGSOC and TNBC by far, potentially or possibly pathogenic variants in DNA repair were detected in 4 out of 21 tumors from young HGSOC patients, i.e., 19% and in 20 out of 60 patients (33%) in the elderly cohort, including genes involved in homologous recombination repair, such as *RAD50* in young patients as well as *BRCA2*, *FANCA,* and *BARD1*, in elderly patients. Three tumors from elderly patients, but none from young patients, presented potentially pathogenic/possibly pathogenic mutations in additional two genes, besides *TP53*: *BARD1* and *CLK2*; *FANCA* and *TOPBP1;* or *FANCA* and *ATR.* Most *TP53*wt tumors did not present any potentially pathogenic or possibly pathogenic variants in their DNA repair genes, except *ATM*, *POLG,* and *RFC1*, each of which was in three different tumors from elderly patients (Figure 5A,B; Table S4).

In TNBC, excluding *TP53*, potentially pathogenic or possibly pathogenic variants were present in 21 (24%) and 13 (56%) of tumors from young and elderly patients, including HRR genes from 3.5% and 21.7% of the tumors from young (*BRCA2*, *BARD1*) and elderly (*BRCA1*, *BRCA2*, *HELQ*, *RAD54B*) patients, respectively. Samples with concomitant DNA repair variants other than *TP53*, occurred in four samples from young patients (*BRCA2* and *FANCG; HLTF* and *POLQ*; *POLG* and *BARD1; PMS1* and *DDB1*) and in four samples from elderly patients (*ATM*, *BRCA1* and *POLQ*; *BRCA1* and *RAD23B*; *CENPE*, *FANCD2,* and *RAD54B)*, including one sample with 10 potentially pathogenic/possibly pathogenic DNA repair genes (Figure 5C,D; Table S4).

## 5. Discussion

We compared two tumor types that share similar molecular features. As seen in other HGSOC and TNBC studies, *TP53* was the most commonly mutated gene in both age groups [23,34,61,62]. The previously cited studies showed a similar *TP53* frequency (~70–90%) compared to the ratio that we observed in this study. In the present series, the frequency of transversions in young TNBC patients was 62%, which was higher when compared to the elderly patients as well as to HGSOC from both age groups, which may be associated with the presence of mutational signature 13. However, this result should be reevaluated because the analysis was not based on raw sequencing data, as described in the Methods section. Mealey and colleagues reported a higher frequency of transversions in younger breast cancer patients, but this difference was discrete (Tv: 53%; Ti: 47%) and was thus probably not statistically significant (no statistical analysis of this type was reported). Chen and colleagues reported a similar proportion of transitions and transversions in young TNBC patients (~50%/50%) [63,64].

The median number of coding variants was higher in TNBC compared to HGSOC from both age groups, and the median number of potentially pathogenic/possibly pathogenic variants per sample was higher in TNBC from elderly patients compared to the number of pathogenic/possibly pathogenic variants found in other groups. We could not find any other studies in young and elderly TNBC and HGSOC patients reporting similar results in terms of variant classification, although Mealey and colleagues and Azim and colleagues reported a higher number of coding variants in elderly breast cancer patients compared to young age groups [64,65]. As previously reported, somatic mutations other than in *TP53* are rare in HGSOC [36,66]. In all four groups, the majority of the samples presented at least one affected oncogene in association with at least one affected tumor suppressor gene (we also considered these as a combination: an oncogene or TSG with a dual-role gene or two or more dual-role genes). All affected elderly TNBC patients presented an association between OG and TSG. 

The predominance of mutational signature 3, which is related to homologous repair defects, was previously reported in both cancer types. In breast cancer samples, mutational signature 3 was also reported to be the most frequent in both young and elderly patients [34,64] as well as in HGSOC in general [67,68]. In addition, a similar signature pattern (1, 3, and 13) was described by Chen and colleagues in young TNBC patients, similar to what has been reported in the present manuscript [63]. Despite that, we found a low frequency of somatic mutations in homologous repair-related genes in both breast and ovarian cancers, although this is not unexpected. In accordance with this, there are studies showing that most alterations in HR-related genes are germline, epigenetic, or CNVs [23,25,36,61].

Independent of age at diagnosis, signature 3 was also the most prevalent in HGSOC patients. In ovarian cancer, signature 3 is the most prevalent in both primary tumors as well as in distant metastatic sites [66,68]. The present results are also in accordance with Yang and colleagues (2018), who explored the somatic characteristics in HGSOC patients and reported that the majority of young patients were mainly represented by mutational signature 3 and that the most commonly altered gene was *TP53* [69].

Multiple studies suggest that the Ras pathway may influence the behavior of TNBC and HGSOC. For instance, Zhu and colleagues recently reported a correlation between Ras/ERK upregulation and chemoresistance in TNBC [70,71]. In the present series, we could not find any differences in the mutation rate of the Ras signaling pathway-related genes while comparing age and cancer type groups. Even though alterations in the RAS and PIK3CA pathways are mostly related to low-grade serous ovarian carcinomas [72,73,74], we were able to detect mutations in those genes in 14.3% and 15% of young and elderly HGSOC, respectively. *NF1*, which was altered in 5%, and 9.5% of young and elderly patients in the currently presented cohort, usually appears as one of most mutated genes in HGSOC analysis and occurs at higher frequencies of 16%-17% when age is not taken into account [62,75]. However, *NF1* may also be altered through structural variants [66], which were not assessed in this study.

Among all of the genes, *KMT2C* was the only one to be more frequently mutated in TNBC from elderly patients than it was in TNBC from young patients, in the present analysis. Similarly, Azim and colleagues reported a higher frequency of *KMT2C* variants in elderly breast cancer patients as well as higher frequencies of *KMT2D*, another member of the lysine methyltransferase family [65,76]. Alterations affecting the lysine-transferase family genes were also observed in high-grade ovarian cancer patients, (*KMT2A* = 2; *KMT2C* = 3), which is in accordance with a prior study (Zhang, 2021), where alterations in *KMT2C* were specifically identified in elderly patients, suggesting that these might be biomarkers that are related to the patient’s age [62,75].

The *CSMD3* gene, a TSG in ovarian cancer (COSMIC; https://cancer.sanger.ac.uk/census; accessed on 1 May 2020), was the second most frequently pathogenically affected gene in HGSOC patients, besides *TP53,* in both age groups. *CSMD3* was also the gene most frequently affected by CNVs in a group of patients with no residual disease after surgery in a HGSOC series [77]. The gene is also in the top 10 mutated genes in the TNBC in the present analysis.

*MUC16* mutations were among the most frequent, both in ovarian and breast cancer samples in the present study. *MUC16*, which encodes for Mucin 16, that contains the CA-125 epitope, a known ovarian cancer marker, was only identified among elderly but not in young HGSOC (8.3%) patients. A similar frequency (6%) was reported recently by Zhang, 2021, but this study did not consider age groups. For TNBC, *MUC16* was identified in 7% of the young patients and 13% of the elderly patients, with no difference between the age and cancer groups [62].

Tumor suppressor genes were the most frequently affected group of known cancer-causing genes in HGSOC, in accordance with what has been reported previously. Even with the high prevalence of *TP53* mutations, it was shown that these tumors present disruptions in another TSG that are either caused by somatic or, more commonly, by structural events [66]. In accordance with this, we observed the presence of mutations in other TSG in the 5 out of 7 and in the 5 out of 11 HGSOC tumors harboring wild-type *TP53* from young and elderly patients, respectively. Likewise, in accordance with that, a CRISPR- analysis in Ovarian Epithelium stem-cells showed that the disruption not of *TP53* alone (which is necessary in many cases, but not sufficient) but the combined disruption of tumor suppressor genes, especially *TP53*, *PTEN,* and *RB1,* are sufficient to induce transformation [78].

Breast cancer behavior has not always been associated with age at diagnosis. In fact, in a recently published study, Aine and colleagues investigated the genetic, transcriptional, and immune characteristics of young TNBC patients. The authors did not find a relevant connection between young patients and TNBC behavior, and they reported the possibility of the differential outcome in this cancer type being influenced by specific genetic signatures rather than by age itself. Although the previous results were strengthened with complementary analysis, the sample size was a limitation, as seen in the present study. The abovementioned statements refer to the need for larger cohort sizes to efficiently investigate the particularities of heterogeneous tumor types in young adult patients [79].

A limitation of our analysis is the absence of complementary molecular data, such as the copy number variation and methylation, which are known to play an important role in ovarian and breast cancer development and progression. Since multiple studies were used, the absence of raw data may result in different variant call pipelines. Moreover, the low number of samples in young high-grade serous ovarian cancer and elderly triple-negative breast cancer patients might limit the power of some of the statistical analysis.

The strength of this study is the individualized search of oncogenes and tumor suppressor genes alone or in combination in tumors from women who are 40 years of age apart and that are related to the dichotomized analysis in well-defined age groups to establish differences between young adults who are aged 18 to 40 years old and elderly women who are aged 70 years old or more, as well as the individualized pathogenicity analysis of each variant.

In summary, mutational signature 3 is more frequently detected in tumors from young patients from both HGSOC and TNBC compared to elderly patients with the same tumor type. In addition, TNBC from young and elderly patients presents a differential mutation signature pattern and TNBC from young women presents a higher mutation rate than it does in elderly women. We also noticed that the frequency of the alterations in cancer-causing genes that were directly involved in the cancer (oncogenes and TSG) was observed in most age groups in both cancer types, although some young adults in both cancer types had no identified gene representing a direct and strong role in their cancer.

The present analysis may contribute to the understanding of these types of tumors; however, further analysis integrating a more representative sample size and other “omics” portraits are necessary to validate the age relevance in different cancer types.

## 6. Conclusions

In conclusion, HGSOC and TNBC are very similar. The median number of mutated CGCs is three in young patients and four and six in elderly, and the main mutational signature is signature 3. At least ⅔ of the tumors presented at least one mutated oncogene in association with tumor suppressor genes.

## Figures and Tables

**Table 1:** Cancer and age groups summaries and comparisons.

		HGSOC			TNBC			Young Patients: TNBC vs. HGSOC	Elderly Patients: TNBC vs. HGSOC
		Young	Elderly	*p*-Value	Young	Elderly	*p*-Value	*p*-Value	*p*-Value
	Median C>T substitutions (%)	37	41	ns	27	35	0.00055	0.00084	ns
	Median *n*. of coding variants/sample	37	44	ns	50.5	61	ns	0.014	0.046
	Median *n*. of MS variants/sample	32	37.5	ns	42.5	53	ns	0.011	0.046
	Median *n*. of potentially pathogenic (FS/SS/NS)/possibly pathogenic variants/sample	14	13.5	ns	17.5	21	ns	ns	0.016
	Median *n*. CGC/sample	3	4	ns	3	6	0.00013	ns	0.001
	Median *n*. CCGD/sample	4	6	ns	6	7	0.044	ns	0.025
Mutational Signatures (%)	1—Age	33.3	41.7	ns	23.3	-	-	-	-
	2—APOBEC (C>T)	-	-			47.8	-	-	-
	3—HR	42.9	25	0.011	50	17.4	1.63 × 10^−6^	ns	ns
	5—Unknown	23.8	33.3	ns	-	-	-	-	-
	6—MMR	-	-	-	-	34.8	-	-	-
	13—APOBEC (C>G)	-	-	-	26.7	-	-	-	-
CGC	CGC combinations (OG and TSG)	14/21 (66.6%)	51/60 (85%)	ns	62/86 (72.1%)	23/23 (100%)	0.0032	ns	ns
	CGC alone (OG or TSG)	5/21 (23.8%)	6/60 (10%)	ns	12/86 (14%)	0/23 (0%)	ns	ns	ns
	DNA repair potentially/possibly	4/21 (19%)	19/60 (32%)	ns	13/86 (15%)	12/23 (52%)	0.0005	ns	ns
	Ras	3/21 (14.3%)	9/60 (15%)	ns	17/86 (19.7%)	8/23 (34.8%)	ns	ns	ns

MS: missense; HR: homologous repair; MMR: mismatch repair; ns: no significance; OG: oncogene; TSG: tumor suppressor gene; FS: frameshift; SS: splice-site; NS: nonsense.

**Table 2:** Top 20 most commonly affected oncogenes (OG) and tumor suppressor genes (TSG) across HGSOC and TNBC (young -Y and elderly -Y). Bold: significant *p*-value.

		HGSOC			TNBC				
Gene	Role in Cancer	Y (*n* = 21)	E (*n* = 60)	*p*-value	Y (*n* = 86)	E (*n* = 23)	*p*-Value	Y-HGSOC vs. Y-TNBC (*p*-value)	E-HGSOC vs. E-TNBC (*p*-value)
*TP53*	OG/TSG	14	50	ns	60	17	ns	ns	ns
*MUC16*	OG	-	5	ns	9	6	ns	ns	ns
*CSMD3*	TSG	2	4	ns	3	2	ns	ns	ns
*PIK3CA*	OG	-	1	ns	4	6	ns	ns	ns
*KMT2C*	TSG	-	3	ns	1	6	**0.006**	ns	ns
*NF1*	TSG	2	3	ns	4	-	ns	ns	ns
*ATM*	TSG	-	3	ns	2	3	ns	ns	ns
*BIRC6*	OG	-	3	ns	3	2	ns	ns	ns
*LRP1B*	TSG	-	2	ns	6	-	ns	ns	ns
*MUC4*	OG	-	-	ns	6	1	ns	ns	ns
*CLTCL1*	TSG	1	2	ns	2	1	ns	ns	ns
*BRAF*	OG	-	1	ns	2	2	ns	ns	ns
*BRCA2*	TSG	-	2	ns	2	1	ns	ns	ns
*CREBBP*	OG/TSG	-	3	ns	1	1	ns	ns	ns
*GRM3*	OG	-	2	ns	3	-	ns	ns	ns
*MED12*	TSG	-	1	ns	2	2	ns	ns	ns
*MET*	OG	-	1	ns	3	1	ns	ns	ns
*NOTCH1*	OG/TSG	-	1	ns	2	2	ns	ns	ns
*RB1*	TSG	1	3	ns	1	-	ns	ns	ns
*ZFHX3*	TSG	-	2	ns	2	1	ns	ns	ns

**Table 3:** Oncogenes and TSGs altered on samples of HGSOC and TNBC from young and elderly patients and their mutational effects.

a—Young HGSOC				
Sample_ID	OG	TSG	OG/TSG	
TCGA-09–1664-01	**KRAS ^++^**	**NF1 ^-^**	-	
TCGA-13–0792-01	CTNND2 ^?^	CIITA ^?^	**TP53 ^-^**	
TCGA-13–0793-01	MECOM ^?^	BAZ1A ^?^	**TP53 ^-^, DAXX ^-^**	
TCGA-13–0884-01	ACVR1 ^?^, TRRAP ^?^	CSMD3 ^?^, SLC34A2 ^?^	BIRC3 *, **TP53 ^-^**	
TCGA-24–1105-01	-	-	**TP53 ^-^**	
TCGA-24–1416-01	**PIK3CA ^++^**	-	**TP53 ^-^**	
TCGA-25–1328-01	-	-	-	
TCGA-25–2404-01	-	ZMYM3 ^?^	**TP53 ^-^**	
TCGA-25–2408-01	-	CDKN1B ^?^	-	
TCGA-29–1688-01	HIP1	-	**TP53 ^-^**	
TCGA-29–1769-01	SETDB1 ^?^	-	RAD21 ^?^	
TCGA-29–2436-01	-	KAT6B ^?^	-	
TCGA-36–2530-01	ERBB2 ^?^	DNMT3A ^?^, IKBK	-	
TCGA-36–2537-01	-	-	**TP53 ^-^**	
TCGA-36–2538-01	-	ERCC3 ^?^, RB1-	**TP53 ^-^**	
TCGA-36–2540-01	-	-	-	
TCGA-59–2363-01	-	CLTCL1 ^?^, CSMD3 ^?^, **NF1 ^-^**	**TP53 ^-^**	
TCGA-61–1725-01	JAK3 ^?^	RAD17 ^?^	**TP53 ^-^**	
TCGA-61–2008-01	-	BLM ^?^, CNTNAP2 ^?^	**TP53 ^-^**	
TCGA-61–2109-01	CHD4 ^?^	FAT1 ^?^	**TP53 ^-^**	
TCGA-61–2611-02	ACKR3 ^?^	-	**TP53 ^-^**	
**b—Elderly HGSOC**				
**Sample_ID**	**OG**	**TSG**	**OG/TSG**	
TCGA-04–1331-01	DDIT3 ^?^	**BRCA2^-^**, KDM5C ^?^, LATS1 ^?^, NF1 ^?^	TBL1XR1 ^?^, **TP53 ^-^**	
TCGA-04–1337-01	-	CDH1 ^?^, FBXW7 ^--^	ELF4 ^?^, **TP53 ^-^**	
TCGA-04–1338-01	MTOR ^?^	CDH11 ^?^, FAT1, KMT2C ^?^	**CREBBP ^-^**, PABPC1 ^?^, **TP53 ^-^**	
TCGA-04–1341-01	CTNND2 ^?^, GRM3 ^?^, PDGFRA ^?^, PREX2 ^?^, TCF7L2 ^?^	CDH10 ^?^, FANCA ^?^, ATR ^?^	**TP53 ^-^**	
TCGA-04–1342-01	KIT ^?^	LZTR1 ^?^, SMARCA4 ^?^	-	
TCGA-04–1347-01	BRAF ^?^, GRM3 ^?^	DROSHA ^?^, FAT1 ^?^	**TP53 ^-^**	
TCGA-04–1351-01	-	-	-	
TCGA-04–1365-01	AFF3 ^?^	**ZFHX3 ^-^**	**TP53 ^-^**	
TCGA-04–1517-01	-	-	**TP53 ^-^**	
TCGA-04–1652-01	-	LRP1B ^?^, PTPRT ^?^	**TP53 ^-^**	
TCGA-09–0364-01	-	CNOT3 ^?^, CNTNAP2 ^?^	**TP53 ^-^**	
TCGA-09–1661-01	DDIT3 ^?^	PBMR1 ^?^, TNFAIP3 ^?^	**TP53 ^-^**	
TCGA-09–1672-01	**KRAS ^++^**	-	-	
TCGA-09–1674-01	LPP ^?^, PIK3CB ^?^	**HNF1A ^-^**, PALB2, **RB1 ^-^**, SETD2 ^?^	**TP53 ^-^**	
TCGA-09–2044-01	CTNNB1 ^?^, MET ^?^	PTPN13 ^?^, RMI2 ^?^, **TSC1 ^-^**	**TP53 ^-^**	
TCGA-10–0933-01	MUC16 ^?^	**RB1-**	RHOA ^?^, **TP53 ^-^**	
TCGA-10–0938-01	NUP98 ^?^, SND1 ^?^	-	**TP53 ^-^**	
TCGA-13–0755-01	MUC16 ^?^	ARID2 ^?^, FEN1 ^?^, GRIN2A ^?^, KMT2C ^?^	**TP53 ^-^**	
TCGA-13–0802-01	-	-	-	
TCGA-13–0888-01	CRTC1 ^?^, MYCN ^?^, RAP1GDS1 ^?^, SOX2 ^?^	BARD1 ^?^, EBF1 ^?^, EP300 ^?^, USP44 ^?^, ZFHX3 ^?^	**TP53 ^-^**	
TCGA-13–0889-01	ERBB2 ^?^	AMER1 ^?^, ATRX ^?^	**TP53 ^-^**	
TCGA-13–1411-01	-	CLTCL1 ^?^, DICER1 ^?^, SMARCB1 ^?^	**TP53 ^-^**	
TCGA-13–1481-01	ARHGAP5 ^?^, GLI1 ^?^, MUC16 ^?^, RARA ^?^	**BRCA2 ^-^**	**TP53 ^-^**	
TCGA-13–1507-01	ATF1 ^?^, SETDB1 ^?^	MLH1 ^?^, PTPRT ^?^	**TP53 ^-^**	
TCGA-20–0991-01	MACC1 ^?^	-	**TP53 ^-^**	
TCGA-20–1686-01	WAS ^?^	CSMD3 ^?^, SLC34A2 ^?^	IRS4 ^?^, **TP53 ^-^**	
TCGA-23–1116-01	-	-	**TP53 ^-^**	
TCGA-23–2641-01	-	-	NOTCH2 ^?^, **TP53 ^-^**	
TCGA-24–0966-01	-	LZTR1 ^?^, N4BP2 ^?^	CREBBP ^?^, TET1 ^?^, **TP53 ^-^**	
TCGA-24–0982-01	-	SMC1A ^?^	**TP53 ^-^**	
TCGA-24–1422-01	CHD4 ^?^, MUC16 ^?^	XPC ^?^	**TP53 ^-^**	
TCGA-24–1552-01	-	-	**TP53 ^-^**	
TCGA-24–1849-01	BRD4 ^?^	GPC5 ^?^, **RB1 ^-^**	BTK ^?^, **TP53 ^-^**	
TCGA-24–2030-01	-	NDRG1 ^?^	BIRC6 ^?^, **TP53 ^-^**	
TCGA-24–2033-01	AFF4 ^?^, GNAS ^?^	DNM2 ^?^, STAG1 ^?^	BIRC6 ^?^, CBLC ^?^, EZH2 ^?^, **TP53 ^-^**	
TCGA-24–2261-01	-	CSMD3 ^?^	**TP53 ^-^**	
TCGA-25–1325-01	MUC16 ^?^	FAT4 ^?^	**TP53 ^-^**	
TCGA-25–1329-01	MTOR *	-	**TP53 ^-^**	
TCGA-25–1634-01	EGFR ^?^	EIF3E ^?^	**TBX3 ^-^**	
TCGA-25–2392-01	FGFR4 ^?^, SRC ^?^	**CDK12 ^--^**, USP44 ^?^	**TP53 ^-^**	
TCGA-25–2393-01	-	MED12 ^?^, RNF43 ^?^	BCORL1 ^?^	
TCGA-25–2399-01	CACNA1D ^?^	**ATM ^-^**, NCOR1 ^?^	**TP53 ^-^**	
TCGA-25–2400-01	WAS ^?^	LPR1B ^?^	**TP53 ^-^**	
TCGA-29–1702-01	KIT	SLC34A2 ^?^	**TP53 ^-^**	
TCGA-29–1761-01	-	CNTNAP2 ^?^, **FANCA ^-^**, PRMD2, SMC1A ^?^	**TP53 ^-^**	
TCGA-29–1771-01	KMT2A ^?^	**NF1 ^-^**	**TP53 ^-^**	
TCGA-29–1774-01	-	DROSHA ^?^, SIRPA ^?^	-	
TCGA-29–1778-01	KMT2A ^?^	-	**TP53 ^-^**	
TCGA-29–2429-01	PSIP1 ^?^, ROS1 ^?^	ATM ^?^, **FLCN ^-^**	-	
TCGA-31–1950-01	PAX3 ^?^	CCD6 ^?^, CTCTCL1 ^?^	CREBBP ^?^, **TP53 ^-^**	
TCGA-36–1575-01	-	KDM5C ^?^	**TP53 ^-^**	
TCGA-36–1576-01	TSHR ^?^	-	-	
TCGA-36–2543-01	RET, UBR5 ^?^	NF1 ^-^	**TP53 ^-^**	
TCGA-42–2587-01	AFF4 ^?^, FCRL4 ^?^, POU2AF1 ^?^	CDC73 ^?^, WNK2	BCL9L ^?^, ESR1 ^?^, **TP53 ^-^**	
TCGA-59–2352-01	A1CF ^?^, SIX1 ^?^, ZEB1 ^?^	CCDC6 ^?^, CNTNAP2 ^?^, CSMD3 ^?^, PRDM1 ^?^	BIRC6 ^?^, **TP53 ^-^**	
TCGA-61–1730-01	ALK ^?^, PLCG1 ^?^	FAT1 ^?^	**TP53 ^-^**	
TCGA-61–1741-01	CHD4 ^?^	-	**DAXX ^-^, TP53 ^-^**	
TCGA-61–1899-01	CDH10 ^?^, PIK3CB ^?^	CSMD3 ^?^, KMT2C ^?^	-	
TCGA-61–2012-01	BCL6 ^?^, SGK1 ^?^	**PHF6 ^-^**, POLG ^?^	NOTCH1 ^?^, TBX3 ^?^	
TCGA-OY-A56Q-01	-	-	**TP53 ^-^**	
**c—Young TNBC**				
**Sample_ID**	**OG**	**TSG**	**OG/TSG**	**Putative Drivers**
BB01_044	-	-	BIRC6 ^?^, KMT2D ^?^, **TP53 ^-^**	-
BB01_074	BCL9 ^?^, CCND3 ^?^	ATM ^?^, DICER1 ^?^, LRP1B ^?^	**CREBBP ^-^**, **TP53 ^-^**	-
BB01_109	ETV1	-	**TP53 ^-^**	-
BB01_126	RET ^?^	-	**TP53 ^-^**	-
BR067	ACK3 ^?^, ETV4 ^?^, GRM3 ^?^, MUC4 ^?^	-	STAT5B ^?^, **TP53 ^-^**	-
BR078	-	-	-	HERC1, HUWE1
BR080	BRAF ^?^	NRG1 ^?^	**TP53 ^-^**	-
BR088	-	-	-	KIF13B
BR091	-	-	**TP53 ^-^**	-
BR097	NR4A3 ^?^, **PIK3CA ^++^**, PTPN11 ^?^	**DICER1 ^--^**, NF1 ^-^	FOXO3 ^?^, **TP53 ^-^**	-
BR100	MET ^?^, PIK3CB ^?^	KMT2C ^?^, LRP1B ^?^	**TP53 ^-^**	-
BR105	RANBP2 ^?^, UBR5 ^?^	LRP1B ^?^	**TP53 ^-^**	-
BR108	MUC16 ^?^	**SMARCA4 ^-^**	BIRC6 ^?^, **TP53 ^-^**	-
BR121	AFF3 ^?^, BRAF ^?^	ATRX ^?^	-	-
BR145	-	ASXL2 ^?^	**TP53 ^-^**	-
BR164	UBR5 ^?^	-	**TP53 ^-^**	-
BR176	ERBB2 ^?^, MUC4	CLTCL1 ^?^	**TP53 ^-^**	-
BR200	PREX2 ^?^, TRRAP ^?^	LRIG3 ^?^	ELF4 ^?^, **TP53 ^-^**	-
BR255	CHST11 ^?^, DNM2 ^?^	BARD1 ^?^, ETV6 ^?^, FHIT ^?^, LPR1B ^?^, POLG ^?^, TET2	-	-
BR301	MUC4	CTLCL1 ^?^	LEF1 ^?^, **TP53 ^-^**	-
BR313	USP6	-	**TP53 ^-^**	-
BR367	STIL ^?^	-	**TP53 ^-^**	-
BR393	-	ZFHX3 ^?^, ZMYM3 ^?^	TET1 ^?^, **TP53 ^-^**	-
BR395	-	-	**TP53 ^-^**	-
BR-M-045	CARD11, HLF ^?^, MUC16 ^?^	LRP1B ^?^	**TP53-**	-
BR-V-022	-	CDH1, LRP1B ^?^	**TP53 ^-^**	-
BR-V-051	-	-	**TP53 ^-^**	-
BR-V-070	-	-	NOTCH1 ^?^	-
PD11326a	-	AXIN2, KAT6B	**TP53 ^-^**	-
PD13627a	-	PTPRC ^?^	-	-
PD18024a	-	-	**TP53 ^-^**	-
PD22036a	-	**NF1 ^-^**	-	-
PD22358a	CDH17 ^?^	POLE ^?^, SETD2 ^?^	-	-
PD23554a	FLI1	BAZ1A ^?^	KMT2D ^?^, **TP53 ^-^**	-
PD23563a	IL6ST ^?^	SPOP ^?^	**TP53 ^-^**	-
PD23566a	HIST1H3B ^?^, MET ^?^	**PTPRD ^-^**	FES ^?^, **TP53 ^-^**	-
PD24182a	MUC4 ^?^, TRIM27	CDX2 ^?^, CSMD3 ^?^	**TP53 ^-^**	-
PD24186a	RAC1 ^?^	-	-	-
PD24191a	CACNA1D ^?^, MUC16 ^?^, MUC4 ^?^	**PTEN ^-^**	-	-
PD24196	-	FAT4 ^?^, PTPN13	**TP53 ^-^**	-
PD24202a	PPM1D ^?^	**NF1 ^-^**	FOXO3 ^?^	-
PD24337a	XPO1 ^?^	CDH11 ^?^, PTPRD ^?^	**TP53 ^-^**	-
PD3905a	**PIK3CA ^++^**	-	**TP53 ^-^**	-
PD4005a	-	ERCC5, PTPN6 ^?^	TBL1XR1 ^?^, **TP53 ^-^**	-
PD4006a	-	-	-	MYT1, NFKB1
PD4107a	ABL2, POU2AF1 ^?^	ARHGEF12 ^?^	NTRK1 ^?^, **TP53 ^-^**	-
PD4833a	UBR5 ^?^	-	BCL9L ^?^, **TP53 ^-^**	-
PD4836a	-	ARID1B ^?^, LARP4B ^?^	**TP53 ^-^**	-
PD5930a	-	**PHF6 ^-^**	RECQL4 ^?^	-
PD5945a	-	-	NFE2L2 ^?^, **TP53 ^-^**	-
PD6406a	PREX2 ^?^	-	-	-
PD6411a	PREX2	-	**TP53 ^-^**	-
PD6413a	-	TET2 ^?^	-	-
PD6722a	CSF3R ^?^	FAZ ^?^, RB1-	**TP53 ^-^**	-
PD9004a	SSX1, TNC	CDC73 ^?^, DNMT3A ^?^, GRIN2A ^?^	**TP53 ^-^**	-
PD9595a	CSF1R ^?^	-	BTK ^?^, **TP53 ^-^**	-
PD9696a	BCL11A ^?^	WNK2 ^?^	CUX1 ^?^, **TP53 ^-^**	-
SA083	-	**ZFHX3 ^-^**	-	-
SA097	BRD4 ^?^, PRDM16 ^?^	CCDC6	**TP53 ^-^**	-
SA208	-	**PIK3R1 ^-^**	-	-
SA220	FOXP1 ^?^, NFATC2 ^?^	ATR ^?^, ROBO2, SMARCA4 ^?^	-	-
SA231	-	-	-	MBD2
SA235	-	BAP1 ^?^	-	-
SA236	FLT3 ^?^, MUC16 ^?^	ARID2 ^?^, IGF2BP2 ^?^	**TP53 ^-^**	-
TCGA-A1-A0SP-01	-	ARID1A ^?^, ZMYM3 ^?^	**TP53 ^-^**	-
TCGA-A2-A04P-01	ERBB3, GRM3 ^?^, **PIK3CA ^++^**, SETDB1 ^?^	ACVR2A ^?^, BRCA2 ^?^, ROBO2 ^?^, TNFAIP3 ^?^	IRS4 ^?^, **TP53 ^-^**	-
TCGA-A2-A0CM-01	KAT6A ^?^	CDH11 ^?^, MGMT ^?^	-	-
TCGA-A2-A3XU-01	-	-	-	-
TCGA-AO-A124–01	CCR7 ^?^, CSF3R ^?^, MAP2K2 ^?^, MUC16 ^?^	BRCA2 ^?^, **CDKN2A-**, EXT2 ^?^, FANCG ^?^	**TP53 ^-^**	-
TCGA-AO-A129–01	BCL11A ^?^, IL6ST ^?^, USP6 ^?^	DDX3X ^?^, KAT6B ^?^	MAP3K13 ^?^, **TP53 ^-^**	-
TCGA-AO-A12F-01	-	-	-	TAF1
TCGA-AR-A0TU-01	AKT3 ^?^, ETV1 ^?^	**GRIN2A ^-^, NF2 ^-^**	-	-
TCGA-AR-A0U1–01	-	BCOR ^?^, RSPO2 ^?^	**TP53 ^-^**	-
TCGA-B6-A0IQ-01	-	SMC1A ^?^	**TP53 ^-^**	-
TCGA-B6-A0RS-01	A1CF ^?^, CHD4 ^?^, MET ^?^, MUC16 ^?^	MED12 ^?^, MUTYH ^?^, ZMYM3 ^?^	**TP53 ^-^**	-
TCGA-B6-A0RT-01	GRM3 ^?^, MUC16 ^?^	-	**TP53 ^-^**	-
TCGA-B6-A0RU-01	ARHGAP5 ^?^	-	POLQ ^?^, **TP53 ^-^**	-
TCGA-B6-A0WX-01	-	SLC34A2 ^?^, ZNRF3 ^?^	EPAS1, **TP53 ^-^**	-
TCGA-BH-A0BL-01	HLF ^?^	CSMD3 ^?^, **LZTR1 ^-^**	**TP53 ^-^**	-
TCGA-BH-A0E0–01	ALK ^?^	-	NOTCH1 ^?^,**RHOA ^-^**, **TP53 ^-^**	-
TCGA-D8-A27F-01	MDM4	**BAP1 ^-^**, CSMD3 ^?^	BIRC6 ^?^, **TP53-**	-
TCGA-E2-A14N-01	MAPK1 ^?^, ROS1 ^?^	ABI1 ^?^, PTCH1 ^?^	**TP53 ^-^**	-
TCGA-E2-A1L7–01	SGK1 ^?^	GPC5 ^?^, PTPRB ^?^	**TP53 ^-^**	-
TCGA-E9-A3QA-01	-	**NF1-**, POT1	-	-
TCGA-OL-A5RW-01	AFF3, CTNND2 ^?^, HNRNPA2B1 ^?^, MUC4, RAF1 ^?^	ATM, AXIN1 ^?^, CBLB ^?^, **MED12 ^-^**	IRS4 ^?^	-
TCGA-OL-A66I-01	RANBP2 ^?^	CYLD ^?^	**TP53 ^-^**	-
**d—Elderly TNBC**				
**Sample_ID**	**OG**	**TSG**	**OG/TSG**	
PD10011a	MPL ^?^	**ASXL1 ^-^, KMT2C ^-^**	**TP53 ^-^**	
PD13298a	RET	CSMD3 ^?^, **KMT2C ^-^**, PTPRB ^?^	NOTCH2 ^?^, **TP53 ^-^**	
PD24333a	**BRAF ^++^, KRAS ^++^**, MUC16 ^?^, PDGFRA ^?^, **PIK3CA ^++^**, ROS1 ^?^	**KMT2C ^-^**	RAD21 ^?^	
PD6047a	-	KMT2C ^?^	**TP53 ^-^**	
PD7067a	FLI1 ^?^, TAL1 ^?^	ATM ^?^, **BRCA1 ^-^**	POLQ ^?^, **TP53 ^-^**	
PD8982a	CALR ^?^, MUC16 ^?^, TRRAP ^?^	ARID1B ^?^, CDH10 ^?^, CLTC ^?^, MED12 ^?^, SPEN ^?^	BIRC6 ^?^, **MAP3K1 ^-^**, NOTCH1 ^?^, POLQ ^?^, **TP53 ^-^**	
PD9575a	BCL3 ^?^, **ERBB3 ^+^**	**PTEN-**, PTPN13 ^?^	**TP53 ^-^**	
PD9584a	H3F3A ^?^, **HRAS ^++^**	-	TBX3 ^-^	
SA031	BRAF ^?^, MET ^?^, NRAS ^?^	DDX3X ^?^, STAG2 ^?^	BCORL1	
SA052	-	**ATM ^-^**	**KDM6A ^-^, NOTCH1 ^-^**	
SA056	ARGHAP5 ^?^, ERBB4 ^?^, **PIK3CA ^++^**	-	**TP53 ^-^**	
SA106	FCRL4, HIF1A	**BRCA2-, **CAMTA1, CSMD3 ^?^, FLCN ^?^, SPEN ^?^	BCL9L ^?^, **TP53 ^-^**	
TCGA-A2-A1G1–01	-	-	**CREBBP ^-^**, **TP53 ^-^**, MAP3K1 ^?^	
TCGA-AC-A2BK-01	-	MYH9 ^?^, PTPRD ^?^	**KMT2D ^-^, TP53 ^-^**	
TCGA-AR-A1AJ-01	-	**PTEN ^--^**	**TP53 ^-^**	
TCGA-BH-A0WA-01	IL6ST ^?^	AXIN1 ^?^, BRCA1 ^-^	TP53 ^-^	
TCGA-BH-A18G-01	ARHGAP5 ^?^, BCL9 ^?^, ERBB3 ^?^, MAML2 ^?^, MUC16 ^?^, MUC4, TRIM27 ^?^, ZEB1	ARHGAP26 ^?^, ARHGEF10L ^?^, **ARID1B ^-^**, ASXL2 ^?^, **ATM ^-^**, ATRX ^?^, BCOR, CARS ^?^, CCNB1IP1 ^?^, CDH10 ^?^, CLTCL1 ^?^, CNOT3, CYLD ^?^, DICER1 ^?^, EP300 ^?^, ERCC3 ^?^, ERCC5 ^?^, ETV6 ^?^, IGH2BP2 ^?^, KDM5C ^?^, KMT2C ^?^, MLF1 ^?^, MYH9 ^?^, **PPP2R1A ^-^**, TET2, ZFHX3 ^?^	FES ^?^, KDM6A ^?^, KMT2D ^?^, MAP3K1 ^?^, QKI ^?^	
TCGA-BH-A1F0–01	SGK1 ^?^, STAT3 ^?^	ASXL2 ^?^, GPC5 ^?^, KMT2C ^?^	-	
TCGA-BH-A1FC-01	CTNNA2 ^?^, DDX5 ^?^, TCF7L2 ^?^, TEC ^?^	CLTC ^?^, NCOR1 ^?^	**TP53 ^-^**	
TCGA-C8-A12K-01	-	ATP2B3 ^?^, IKZF1 ^?^, STK11 ^?^	FES ^?^, IRS4 ^?^, **TP53 ^-^**	
TCGA-C8-A131–01	**KRAS ^++^, PIK3CA ^++^**	NTHL1 ^?^, PTPRD ^?^, SETD2 ^?^	**TP53 ^-^**	
TCGA-D8-A1JK-01	CTNNA2 ^?^, KATA ^?^, **PIK3CA ^++^, STAT3 ^+^**, TNC ^?^	ASXL1 ^?^, FANCD2 ^?^, FAT4 ^?^, NCOR1 ^?^	**TP53 ^-^**	
TCGA-E2-A1LK-01	CACNA1D ^?^	EP300 ^?^, LARP4B ^?^, MED12 ^?^	**KDM6A ^-^, TP53 ^-^**	

Oncogenes and TSG (in accordance with Cancer Gene Census—Cosmic—available at https://cancer.sanger.ac.uk/census, accessed on 1 May 2020) altered on samples of young and elderly HGSOC and TNBC patients. OG: oncogene; TSG: tumor supressor gene; OG/TSG: genes with dual role. Green-highlighted genes: genes with benign variants. Red-highlighted genes: Ras pathway-related gene; purple-highlighted gene: RB pathway-related gene. Variant effect (OncoKB): ^++^: gain-of-function; ^+^: likely gain-of-function; ^--^: loss-of-function; ^-^: likely loss-of-function; *: likely neutral; ^?^: variant or gene not curated or not found in the literature. Bold: genes with annotated likely loss and gain-of-function or just loss or gain-of-function in OncoKB.

## Data Availability

Mutational and clinical data from two of the five studies (Cancer Genome Atlas Network, *Nature*, 2012; Nik-Zainal S. et. al., *Nature*, 2016) were downloaded from the International Cancer Genome Consortium—ICGC data portal (https://dcc.icgc.org/; realease date: January 2020). The other studies had their data downloaded from the supplementary data from their respective original articles [25,32,33,34,35].

## Supplementary Materials

The following are available online at https://www.mdpi.com/article/10.3390/cells10123586/s1, Figure S1. Mutational signatures. Figure S2. Number of non-synonymous coding variants per sample. Figure S3. Overlapped frequently affected genes classified according to its variant type. Figure S4. TP53 variants. Table S1. Summaries of variant types and frequencies and clinical data of the samples selected in the article. Table S2. Comparison between TI and TV across age groups and cancer types. Table S3. Comparison between the different cancer cohorts in the TCGA project and the cohorts in this study. Table S4. Mutational data collected from multiple studies and datasets for both tumor types that were analyzed. Table S5. Genes catalogued at the Cancer Gene Census and altered in the samples selected for this study. Table S6. Number of HGSOC and TNBC young and elderly patients with tumors presenting a cancer gene census. Table S7. Most frequently altered genes in all summed groups. Genes in bold are classified as dual roles in cancer (oncogene and TSG); genes highlighted in dark blue are classified as TSG. Table S8. HGSOC young patients: gene ontology (GO) aspect (molecular function, MF; biological process, BP) enriched in genes affected with potentially pathogenic or possibly pathogenic variants. Table S9. HGSOC elderly patients: gene ontology (GO) aspect (molecular function, MF; biological process, BP) enriched in genes affected with potentially pathogenic or possibly pathogenic variants. BP: Biological process; MF: molecular function. Table S10. TNBC young patients: gene ontology (GO) aspect (molecular function, MF; biological process, BP) enriched in genes affected with potentially pathogenic or possibly pathogenic variants. Table S11. TNBC elderly patients: Gene ontology (GO) aspect (molecular function, MF; biological process, BP) enriched in genes affected with potentially pathogenic or possibly pathogenic variants.
