# Supplementary material for: Somatic Mutational Profile of High-Grade Serous Ovarian Carcinoma and Triple-Negative Breast Carcinoma in Young and Elderly Patients: Similarities and Divergences
Source: Cells. 2021 Dec 20;10(12):3586. doi: 10.3390/cells10123586 (PMC8700427; doi:10.3390/cells10123586)
Supplement: Supplementary file 1 [file cells-10-03586-s001.zip › cells-1349475-supplementary/Supplementary Figures.pdf]

Suppementary Figures

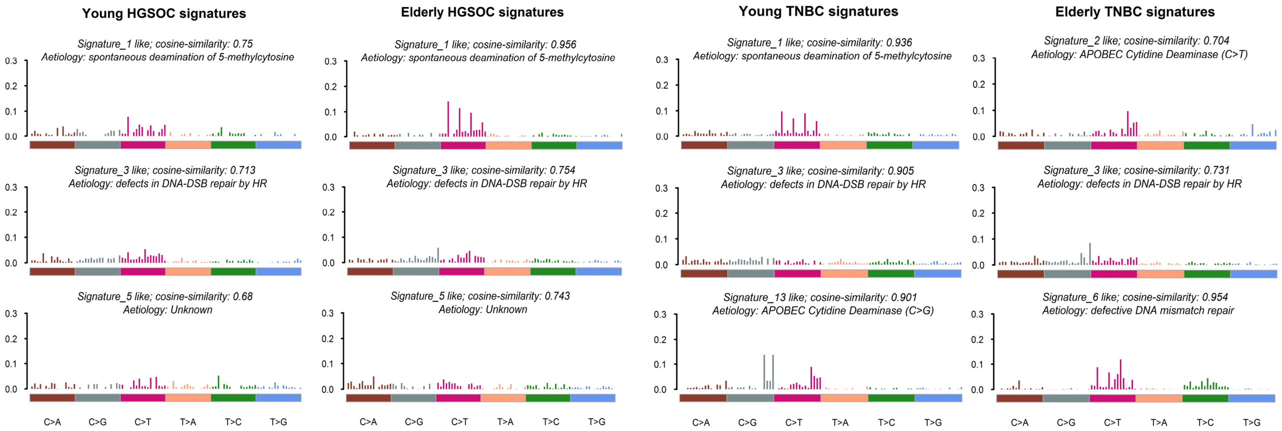

Figure S1. Mutational Signatures. For each signature, a barplot of the frequency distribution of single base substitutions in the trinucleotide context is shown.

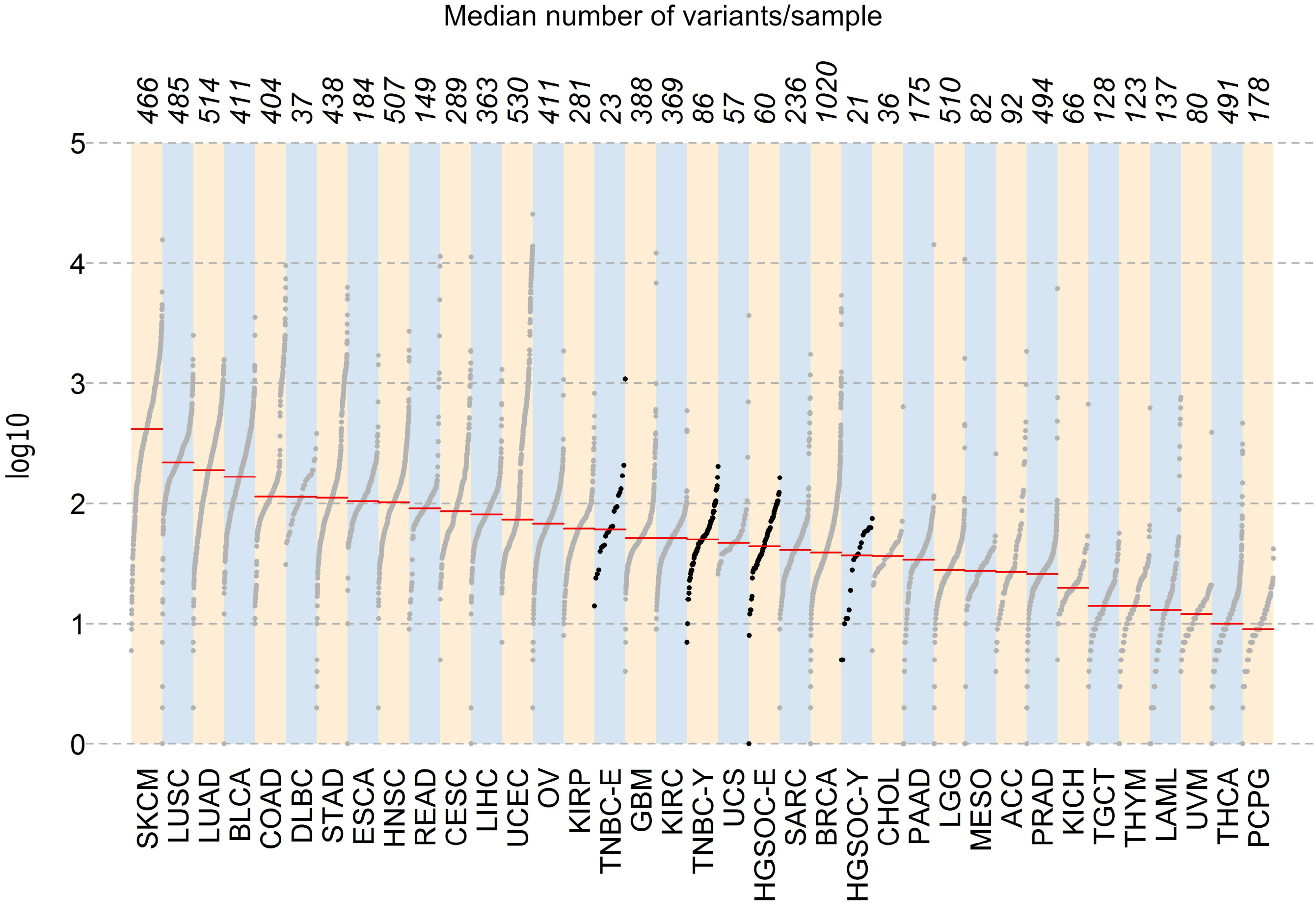

Figure S2. Number of non-synonymous coding variants per sample. The dot plot shows the distribution and median number (red line) of non-synonymous coding variants per sample in TCGA cohorts and those displayed in this work. Each point represents a patient (patients from our selected series are highlighted in bold). Y-axis: Number of non-synonymous coding variants (log10); X-axis: bottom: cohorts names; top: number of patients. HGSOC-Y: Young HGSOC patients; HGSOC-E: Elderly HGSOC patients; TNBC-Y: Young TNBC patients; TNBC-E. HGSOC-Y vs TCGA OV: p=4.3 × 10^{-6}; HGSOC-E vs TCGA OV: p=1.8 × 10^{-5}; TNBC-Y vs TCGA BRCA: p=0.019; TNBC-E vs TCGA BRCA: p=0.0091.

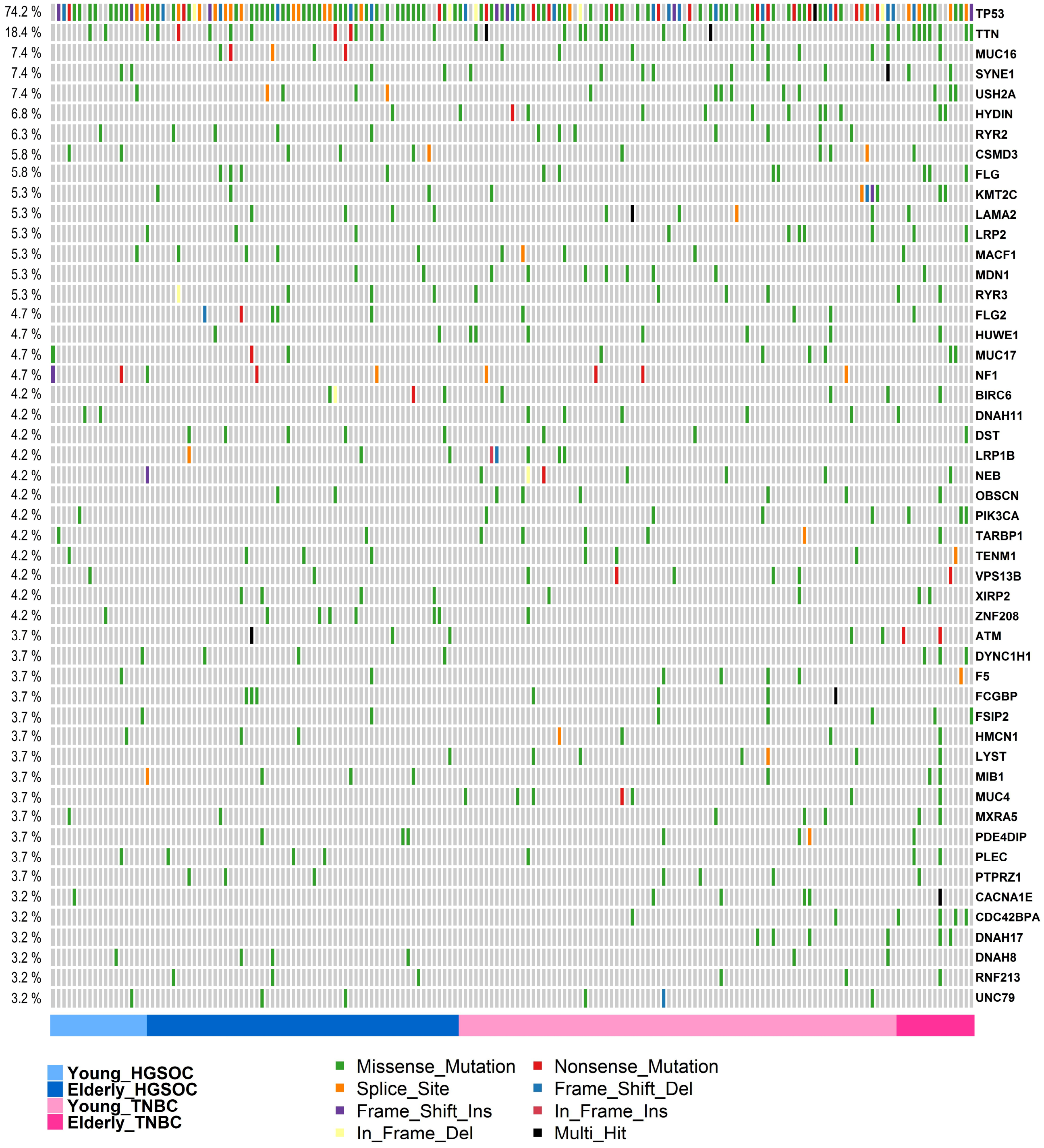

Figure S3. Overlapped frequently affected genes classified according to its variant type. Each column represents a patient and each line a gene (177 samples shown).

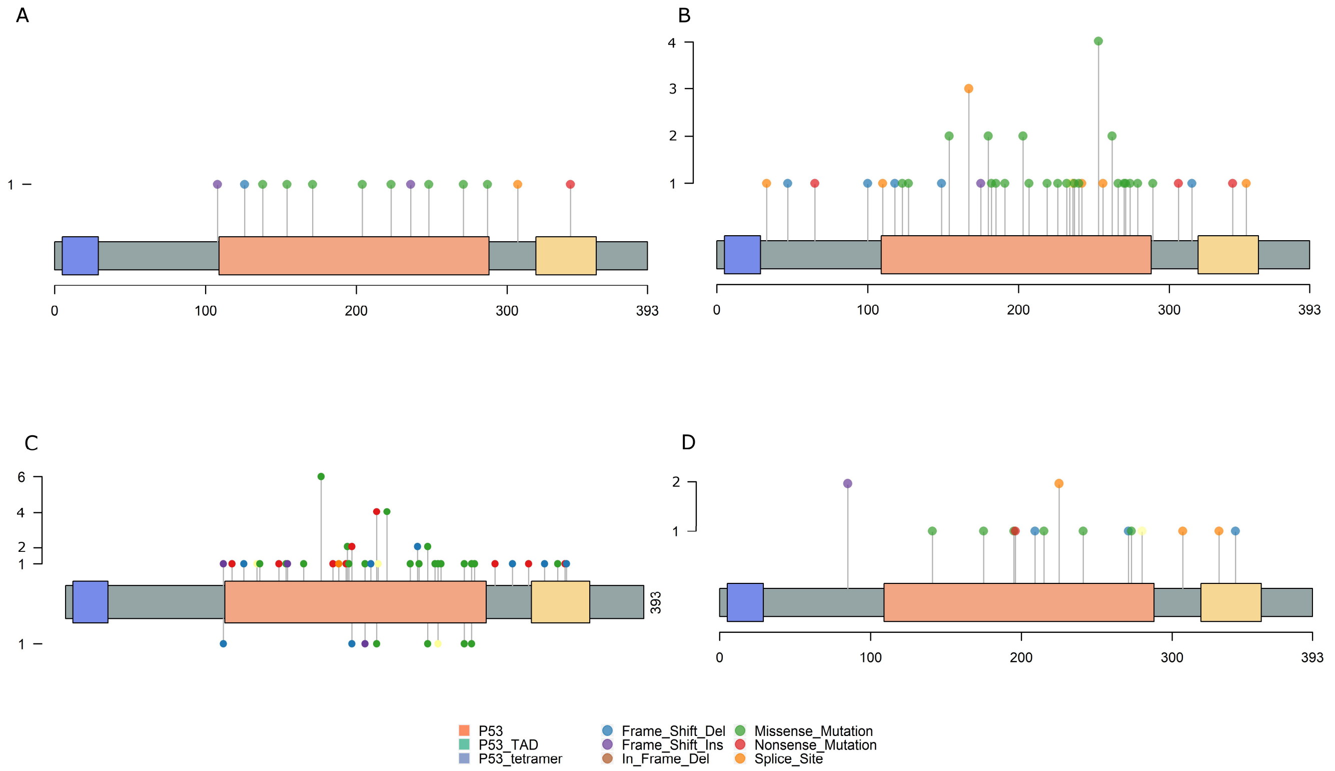

Figure S4. *TP53* variants. (**A**) Young HGSOC patients; (**B**) Elderly HGSOC patients; (**C**) Young TNBC patients; (**D**) Elderly TNBC patients. Y-axis: number of variants; X-axis: amino acid position and domains; P53: P53 DNA-binding domains; P53 Tetramer: P53 tetramerization motif; P53 TAD: P53 transactivation motif.
